# Chest Computed Tomography Findings in HIV-Infected Individuals in the Era of Antiretroviral Therapy

**DOI:** 10.1371/journal.pone.0112237

**Published:** 2014-11-19

**Authors:** Emily Clausen, Catherine Wittman, Matthew Gingo, Khaled Fernainy, Carl Fuhrman, Cathy Kessinger, Renee Weinman, Deborah McMahon, Joseph Leader, Alison Morris

**Affiliations:** 1 Department of Medicine, School of Medicine, University of Pittsburgh, Pittsburgh, Pennsylvania, United States of America; 2 Department of Radiology, School of Medicine, University of Pittsburgh, Pittsburgh, Pennsylvania, United States of America; 3 Department of Immunology, School of Medicine, University of Pittsburgh, Pittsburgh, Pennsylvania, United States of America; 4 Kaiser Permanente, Denver, Colorado; Fundacion Huesped, Argentina

## Abstract

**Background:**

Chest radiographic abnormalities were common in HIV-infected individuals in the pre-combination antiretroviral therapy era, but findings may differ now due to a changing spectrum of pulmonary complications.

**Methods:**

Cross-sectional study of radiographic abnormalities in an HIV-infected outpatient population during the antiretroviral therapy era. Demographics, chest computed tomography, and pulmonary function tests were obtained in HIV-infected volunteers without acute respiratory illness from the University of Pittsburgh HIV/AIDS clinic. Overall prevalence of radiographic abnormalities and potential risk factors for having any abnormality, nodules, or emphysema were evaluated using univariate and multivariable analyses.

**Results:**

A majority of the 121 participants (55.4%) had a radiographic abnormality with the most common being emphysema (26.4%), nodules (17.4%), and bronchiectasis (10.7%). In multivariate models, age (odds ratio [OR] per year  = 1.07, 95% confidence interval [CI] 1.04–1.14, p<0.001), pneumonia history (OR  = 3.60, 95% CI  = 1.27–10.20, p = 0.016), and having ever smoked (OR  = 3.66, p = 0.013, 95% CI  = 1.31–10.12) were significant predictors of having any radiographic abnormality. Use of antiretroviral therapy, CD4 cell count, and HIV viral load were not associated with presence of abnormalities. Individuals with radiographic emphysema were more likely to have airway obstruction on pulmonary function tests. Only 85.8% participants with nodules had follow-up imaging resulting in 52.4% having stable nodules, 23.8% resolution of their nodules, 4.8% development of a new nodule, and 4.8% primary lung cancer.

**Conclusions:**

Radiographic abnormalities remain common in HIV-infected individuals with emphysema, nodules, and bronchiectasis being the most common. Age, smoking, and pneumonia were associated with radiographic abnormalities, but HIV-associated factors did not seem to predict risk.

## Introduction

With advancements in antiretroviral therapy (ART), chronic co-morbidities of HIV have now become more common as the life expectancy of those with HIV has increased [1]–[4]. The spectrum of lung diseases has changed, with conditions such as chronic obstructive pulmonary disease (COPD), pulmonary hypertension, and lung cancer becoming more important in the current HIV-infected population [5]–[9].

Prior to the introduction of ART, studies in HIV-infected individuals reported high prevalence of radiographic abnormalities such as nodules, ground-glass opacities, and intrathoracic lymphadenopathy [10]–[15]. Many abnormalities were related to either past or chronic infections in patients with and without respiratory complaints [16]. Findings on computed tomography (CT) have been used to accurately diagnose pulmonary complications of AIDS [15]. During the current era of HIV, radiographic findings may differ in light of the decreasing incidence of pulmonary infections, but few studies have re-examined radiographic findings on chest CT examination in the contemporary population [17]. Given the frequency of chronic respiratory complaints in HIV-infected populations [18], [19] and the high prevalence of CT scanning [20], understanding radiographic manifestations of HIV is important for patient care.

We performed a cross-sectional study of radiographic abnormalities in an HIV-infected outpatient population without acute respiratory illness. Pulmonary function testing was also performed to determine the relationship between visual assessment of CT exams and physiologic measurements of COPD [21]–[23]. The objective of the study was to assess the prevalence and nature of radiographic abnormalities on chest CT examinations in an HIV-infected cohort during the ART era and to identify risk factors for common findings.

## Materials and Methods

### Participants

Participants were recruited from the University of Pittsburgh HIV/AIDS Clinic. All participants were recruited from an outpatient clinic setting and were not admitted inpatient to a hospital. Details of this cohort have been previously reported [24]. Individuals with documented HIV infections who were 18 years of age or older were recruited from the clinic between July 1, 2007 and September 30, 2010. Participants were able to have chronic respiratory symptoms such as a chronic cough but were excluded if they had an acute change in these symptoms such as worsening cough, increased shortness of breath, etc. within the previous 4 weeks of their visit date. The chronic respiratory symptoms queried in our survey included details about cough, phlegm production, wheezing, and shortness of breath. Individuals were excluded if they reported acute change in respiratory symptoms or fever in the 4 weeks prior to study entry or had contraindication to performing chest CT scan or pulmonary function tests (PFTs). Patients were able to have chronic respiratory symptoms such as a chronic cough phlegm production, wheezing, and shortness of breath. Written informed consent was obtained, and the University of Pittsburgh Institutional Review Board approved the protocol.

### Data collection

Demographic and clinical data were obtained during participant interviews and review of medical record. Collected data included age, gender, smoking history, intravenous drug use (IVDU), self-report of pneumonia history, and self-report of current use of antiretrovirals. Extensive smoking history was obtained for any participant that was ever a smoker, which was defined as having smoked more than 100 cigarettes in their lifetime. Information regarding IVDU history, marijuana use, or crack cocaine use was obtained by self-report of ever user, use within 6 months prior to study date, and use within one week prior to study date. Medical records from within 6 months preceding the study were reviewed to record the most recent CD4+T-lymphocyte cell count and plasma HIV-RNA levels of participants. All participants answered a standardized respiratory questionnaire [25].

### Chest computed tomography

Non-contrasted CT scans were acquired at 100 mAs and reconstructed using both the GE “bone” and “standard” kernels at 0.625 mm thickness at end-inspiration. Chest CT scans were performed for research purposes, not clinical indications. Using standardized definitions [26], each CT scan was visually reviewed and rated by a radiologist and a pulmonologist. Each reader was blinded to participant characteristics. In cases of differing CT scan interpretation, a third reader reviewed the CT scans in order to resolve discrepancies. Radiographic abnormalities were defined as presence of bulla, cysts, nodules, bronchiectasis, ground glass opacities, emphysematous changes, pleural disease, consolidation, honeycombing, mosaic attenuation, and reticular abnormality. To quantify the presence of emphysema, the percentage of the lung voxels associated with emphysema was computed using the density mask technique [27].

### Pulmonary function testing

Participants performed pre- and post-bronchodilator spirometry according to standard American Thoracic Society (ATS) standards [28]. Spirometry predicted values were calculated by Hankinson equations [29]. Neas prediction equations were used to calculate diffusing capacity for carbon monoxide (DL_CO_) with adjustments for hemoglobin and carboxyhemoglobin levels obtained at time of pulmonary function testing [30].

### Statistical analysis

Prevalence of each radiographic abnormality was determined, and risk factors for having any defined abnormality were evaluated using univariate and multivariable analyses. Variables were transformed as necessary to approximate normality. Because non-calcified nodules and emphysema were common and clinically important, in-depth analyses of each of these findings were also performed. For nodules, we determined the median number and size of nodules. For visually assessed emphysema, we determined distribution, extent (scoring 0 = <5%, 1 = 5–10%, 2 = 11–25%, 3 = 26–50%, 4 = 51–75%) and type of emphysema (panacinar, centrilobular, etc.). Bivariate analysis of factors associated with nodules or emphysema was performed using t-tests, rank sum test, chi-square, or Fisher's exact. The relationship of visually assessed emphysema to pulmonary function variables including forced expiratory volume in one second (FEV_1_) percent predicted, forced vital capacity (FVC) percent predicted, FEV_1_/FVC ratio, and DLco percent predicted were determined using t-tests. The percentage of the lung voxels less than -910 Hounsfield units (HU) and less than -950 HU was evaluated in relation to presence of any emphysema using t-tests or rank-sum.

## Results

### Description of cohort

A total of 121 participants were included in the analysis. The majority (67.8%) was male (Table 1). Average age was 45.1 years with a range from 21–72 years. The majority of participants (85.1%) were currently taking ART and median time of HIV infection was 15.8 years with a range  = 1.60–29.6 years. There was a predominance of smokers (80.2%) and only 4 participants reported ever using intravenous drugs. About a quarter (23.1%) of the sample reported having had at least one prior episode of pneumonia requiring treatment and a smaller percentage reported a history of TB (9%). No participants had a history of pleural effusion.

**Table 1:** Description of Cohort and Relationship of Clinical Variables to Radiographic Abnormalities, n = 121.

Characteristic		Without abnormality n = 54	With abnormality n = 67	p-value <0.05
Male, n (%)	82 (67.8)	28 (51.9)	54 (80.6)	0.480
Age, mean years (SD)	45.1 (9.8)	42.5 (9.1)	47.2 (9.9)	0.009
African-American, n (%)	54 (44.6)	28 (51.8)	26 (41.8)	0.480
Ever smoker, n (%)	97 (80.2)	38 (70.3)	59 (88.1)	0.015
Currently taking ART, n (%)	103 (85.1)	43 (79.6)	60 (89.6)	0.127
CD4 count, median cells/µl (range)	537 (24–1798)	520 (21–1798)	561 (24–1493)	0.913
Viral load, median copies/ml (range)	49 (49–1,960,000)	49 (49–1,960,000)	49 (49–522,000)	0.098
IVDU, n (%) (of 101)	4 (4.0)	0 (0)	4 (6.0)	0.073
Previous pneumonia, n (%)	28 (23.1)	8 (14.8)	20 (29.9)	0.046

n = number, SD  =  standard deviation, ART  =  anti-retroviral therapy, IVDU  =  intravenous drug us.

### Radiographic abnormalities

**Figure 1:**
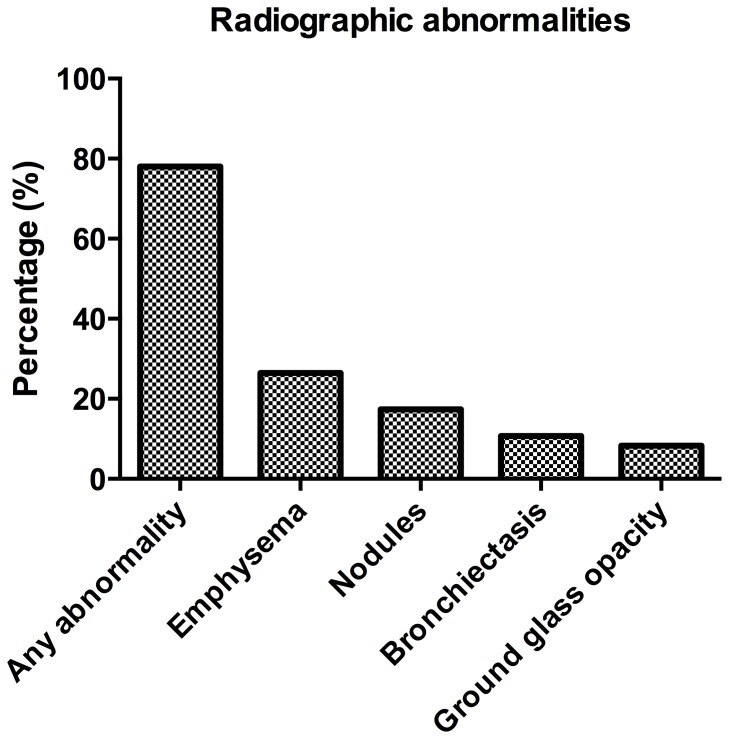
Frequency of radiographic abnormalities visualized among all participants. None of the participants had evidence of consolidation, mosaic perfusion, or honeycombing.

**Figure 2:**
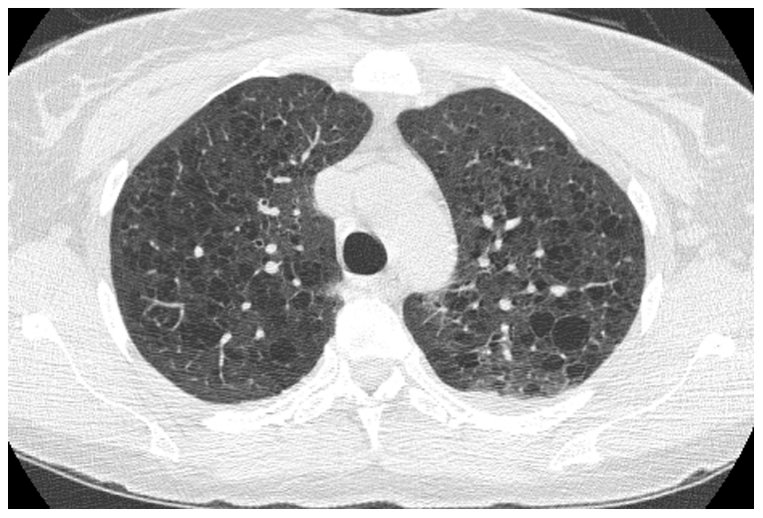
Selected CT image showing emphysema in HIV patient.

A majority of participants (55.4%) had a visually detected abnormality on chest CT exam (Figure 1). Overall, emphysema was the most common finding (26.4%) (Figure 2) followed by nodules (17.4%). Bronchiectasis and ground glass opacities were also fairly common (10.7% and 8.3% respectively). None of the participants had evidence of adenopathy, consolidation, mosaic perfusion, or honeycombing. Overall, a third reviewer was needed to resolve discrepancies on average 6.9% of cases with the most common discrepancies in question being the presence of bronchiectasis (17.4%), ground glass opacities (15.7%), and emphysema (14.0%).

### Predictors of any abnormality

Individuals with an abnormality on CT scan were significantly older on average than those without abnormalities (47.2 years versus 42.5 years, p<0.001), significantly more likely to have smoked in the past, and significantly more likely to have a history of previous pneumonia (Table 1). Only one participant reported known history of *Pneumocystis pneumonia*, so this was not included as a predictor. Subjects with abnormalities on CT examination were more likely to have an undetectable HIV viral level, but this relation was not statistically significant. There was no relationship of CD4 cell count to presence of abnormalities. Subjects with visualized abnormalities on CT chest were not significantly more likely to have chronic respiratory complaints such as chronic cough, phlegm, wheezing, and shortness of breath. In multivariate models, age (odds ratio [OR]  = 1.07 for every year increase, 95% confidence interval [CI] 1.04–1.14, p<0.001) and having smoked (OR  = 3.66, p = 0.013, 95% CI  = 1.31–10.12) were significant predictors of a radiographic abnormality.

### Nodules

Pulmonary nodules were common (17.4%). The majority of participants with nodules had a solitary nodule (range of number of nodules  = 1–14). Median length of the long axis of nodules was 6 mm (range 2–30 mm). Smokers were somewhat more likely to have nodules (19.6% versus 8.3% of non-smokers, p = 0.19), and individuals with nodules tended to have visualized emphysema (25.0% with emphysema versus 14.6% without nodules, p = 0.18), but neither comparison reached significance. Of those participants with pulmonary nodules, 14.2% did not obtain follow-up. Of participants who underwent follow-up chest CT exam, 52.4% had stable nodules without a specific diagnosis or additional work-up and 23.8% had resolution of their nodules. One participant out of the cohort was diagnosed with primary lung cancer during the timeframe of our study. On average, the length between initial and follow-up chest CT exam was 17 months.

### Analyses of emphysema

Most participants with emphysema on visual assessment had upper lobe disease (n = 25, 78.1%), but mid- and lower lung emphysema was also common, seen in 56.3% and 40.6%, respectively. No participants had pan-lobular emphysema. The majority of subjects with emphysema on visual assessment had mild emphysema (1–10% of lung involvement). Subjects with visually assessed emphysema were significantly older, more likely to have smoked, and somewhat more likely to have ever used intravenous drugs. They were not more likely to report chronic respiratory complaints such as chronic cough, phlegm, wheezing, and shortness of breath (data not shown). Multivariate models of risk factors for emphysema demonstrated that age (OR  = 1.15 for every year increase, p<0.001, 95% CI  = 1.07–1.24) and having smoked (OR  = 29.1, p = 0.006, 95% CI  = 2.68–315.18) were significant independent predictors of emphysema.

**Figure 3:**
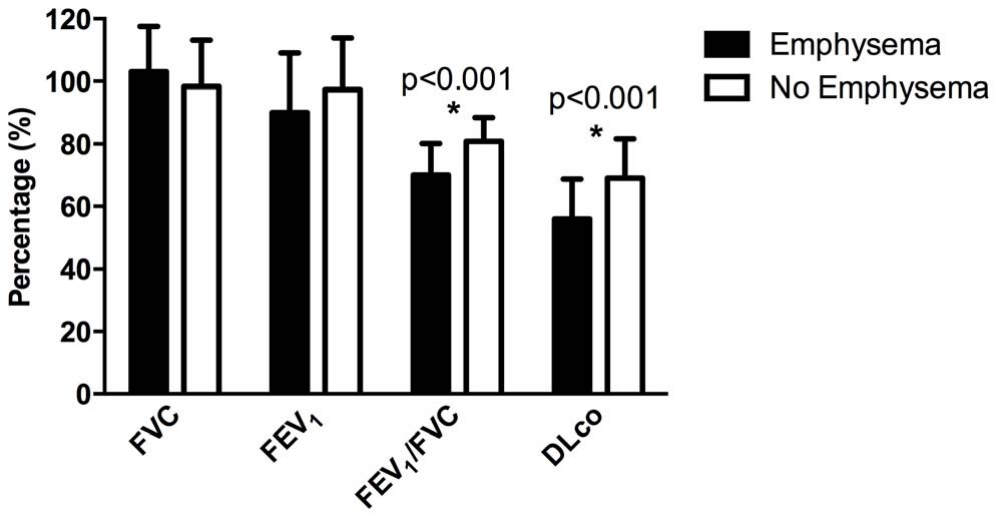
Percentage of Individuals with visually assessed radiographic emphysema. FEV_1_  =  forced expiratory volume; FVC  =  forced vital capacity; DL_CO_  =  diffusing capacity for carbon monoxide.

**Figure 4:**
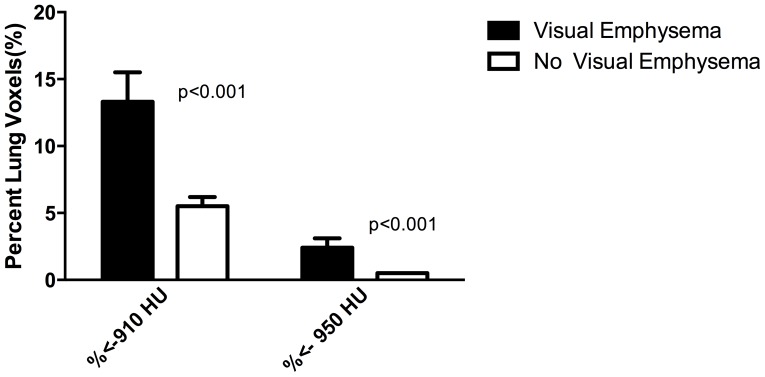
Relation between visually and quantitatively assessed emphysema as measured by percentage of lung voxels less than -910 or -950 HU.

Individuals with visually assessed radiographic emphysema were more likely to have airflow obstruction and decreased DLco (Figure 3). They also had more quantitative emphysema as measured by either percentage of lung below -910 or -950 HU (Figure 4). Medical record review of participants with any visually assessed emphysema revealed only 24% had been diagnosed with COPD by ICD-9 code.

## Discussion

Our study evaluates the prevalence and spectrum of abnormalities on chest CT examination in an HIV-infected population in the current ART era. A majority of participants were found to have a radiographic abnormality; emphysema, nodules, and bronchiectasis were the most common. No adenopathy was present among our participants which may reflect that our cohort is relatively healthy given the study is limited to an outpatient population that regularly engages in healthcare with a designated HIV clinic. Significant predictors of any radiographic abnormality were age or tobacco exposure. HIV-specific predictors such as CD4 cell count and HIV viral level were not significantly associated with radiographic abnormalities.

Overall, our study population had a strikingly high prevalence of radiographic abnormalities, although no HIV-uninfected control group was available for comparison. Various studies done prior to the ART era also showed chest radiographic abnormalities were common in HIV-infected populations [11]–[15]. One study from the pre-ART era that examined a cohort of AIDS patients referred for chest CT examination found that 72% had active disease with *Pneumocystis* pneumonia, Kaposi sarcoma, tuberculosis, and pyogenic or fungal infections being the most common diagnoses [15]. Diseases were characterized by CT image findings such as ground glass opacities in patients with *Pneumocystis* or peribronchovascular nodules with Kaposi sarcoma [14], [15]. In contrast, our study found emphysema and nodules to be the most common abnormalities. This difference illustrates the changing trend of pulmonary disease in HIV-infected populations from acute infection to chronic conditions. Of note, these pre-ART studies were comprised mostly of participants who had AIDS and did not specifically evaluate emphysema.

HIV-associated variables may be related to chest CT scan findings. Prior to the ART era, CD4 cell counts <200 combined with intrathoracic lymphadenopathy on imaging predicted Kaposi sarcoma, mycobacterial diseases, and *Pneumocystis* pneumonia [31]. In a follow-up study, no association was found between CD4 cell count and pulmonary nodules possibly because the majority of the study population had low CD4 counts [12]. In contrast, our participants were outpatients, had chest CT examinations performed electively as part of a research study, and had high CD4 cell counts. We found no relationship between CD4 count and radiographic abnormalities.

Nodules are common and clinically important given the increase in lung cancer rates in HIV-infected populations [32], [33]. Prior to the ART era, nodules on chest CT examination were most likely due to cancer, tuberculosis, or Kaposi sarcoma [12], [15]. Follow-up imaging in most of our study's participants showed stable nodules, and only one participant underwent biopsy. Diagnoses in our study include inflammation due to bronchiolitis, unknown etiology, and adenocarcinoma. In a study that examined abnormal CXR findings in HIV-infected populations, abnormal imaging led to invasive diagnostic testing of 79% of participants [16]. Many of the patients in that study, as in our study, did not have acute respiratory symptoms (CXR had been done for non-pulmonary complaints, constitutional symptoms, screening for incarceration), but were found to have occult opportunistic infections. Fewer opportunistic infections in the ART era likely contribute to the tendency for nodules in our cohort to be stable and chronic. Although most nodules were stable over the time period of the study, it is not known if they might eventually be diagnosed as malignant as we may have insufficient length of follow-up. Also, almost 15% did not have further evaluation of their nodules despite recommendations, limiting ability to determine a diagnosis.

The frequency of nodules in this population could have implications for determining utility of lung cancer screening. We did not have an HIV-uninfected group for comparison, but in the general population in our cohort's geographical region, a similar frequency of nodules found on CT imaging has been reported in a study of participants who are former and current smokers [34], [35]. Trials have shown screening for lung cancer using chest CT reduces mortality from lung cancer among smokers deemed at high risk [35], [36]. Given lung cancer tends to be diagnosed at more advanced stages and at a younger age in those infected with HIV [37], separate studies are necessary to establish specific guidelines for screening of HIV-infected populations. One such recent study compared potential harm versus benefit of the use of low dose CT scan for lung cancer screening between asymptomatic HIV-infected and uninfected groups [17]. The study did not find an increased rate of abnormalities leading to follow-up testing in HIV-infected patients, though it did identify that those with CD4 counts <200 may be more likely to have false positive findings and potentially unnecessary invasive testing [17].

Similar to studies in the pre-ART era, emphysema was common in our cohort. Centrilobular emphysema was most common, and the degree of radiographic emphysema was mild, although emphysema might be greater in an older HIV-infected cohort. Although it is difficult to compare different populations, a pre-ART study found a somewhat lower prevalence of radiographic emphysema of 15%, suggesting that emphysema may now be more common [37]; however, pre-ART cohort participants were on average 10 years younger and had less smoking prevalence when compared to the current study, which may also explain this difference. While we were not able to compare our study population to an HIV-uninfected group, the pre-ART study also reported an incidence of emphysema on 2% of the CT exams in their HIV-uninfected participants. A prevalence of emphysema of 42.5% was found among subjects in a more recent study examining high-risk populations due to a substantial smoking exposure history [38].

Age and smoking predicted presence of emphysema, but HIV-associated factors did not correlate with evidence of emphysema on CT exam. This finding is consistent with prior results that showed no difference in number of participants with COPD between those taking or not taking ART [8]. Radiographic findings of emphysema correlated with PFT evidence of obstructive disease and reduced DLco. Only a quarter of the participants with visualized emphysema had been diagnosed with COPD, suggesting that emphysema is likely under-diagnosed in HIV-infected individuals and possibly missed as an important risk factor for lung cancer.

Limitations of the study included that it was a single cohort, had a high prevalence of smoking, antiretroviral therapy use was measured by self-report, pneumonia history was by self-report, and the study lacked an HIV-uninfected control group. Patients enrolled in the study obtain their medical care in different settings making it difficult to verify our facility medical record contains every episode of pneumonia for a given patient. Lack of power may have affected the significance for various associations with radiographic findings. The high prevalence of smoking among the cohort may limit the generalizability of the prevalence of different findings to similar populations, but many HIV-infected groups have a high prevalence of smoking. Intravenous drug use was also relatively uncommon in our cohort, which might also influence the types of abnormalities seen. Although we saw no association of abnormalities with or without ART use, we did not capture individuals starting ART who might have a different spectrum or prevalence of radiographic findings.

We have found that radiographic abnormalities remain common in HIV-infected individuals with emphysema and nodules being the most common findings of clinical relevance. In this outpatient cohort, age and smoking were associated with radiographic abnormalities, but HIV-associated factors such as CD4 cell count and viral load were not associated with radiographic abnormalities. Nodules were a frequent finding, and most were stable or resolved without diagnostic or therapeutic intervention. Our findings indicate that emphysema is common in HIV-infected populations and that visually assessed emphysema strongly correlated with quantitatively assessed emphysema and with lung function.

Some results were previously presented in the form of a poster at the May 2011 American Thoracic Society International Conference in Denver, CO.

## References

[1] UNAIDS (2012) Joint UN programme on HIV/AIDS. Data and Analysis AIDSinfo.

[2] Organization WH (2011) Global HIV/AIDS response – Epidemic update and health sector progress towards universal access. World Health Organization.

[3] Control CfD (2013) HIV in the United States: At a Glance.

[4] May M, Gompels M, Delpech V, Porter K, Post F, et al (2011) Impact of late diagnosis and treatment on life expectancy in people with HIV-1: UK Collaborative HIV Cohort (UK CHIC) Study. BMJ 343: d6016. 21990260 10.1136/bmj.d6016 PMC3191202

[5] Sitbon O, Lascoux-Combe C, Delfraissy JF, Yeni PG, Raffi F, et al (2008) Prevalence of HIV-related pulmonary arterial hypertension in the current antiretroviral therapy era. Am J Respir Crit Care Med 177: 108–113. 17932378 10.1164/rccm.200704-541OC

[6] Sigel K, Wisnivesky J, Gordon K, Dubrow R, Justice A, et al (2012) HIV as an independent risk factor for incident lung cancer. AIDS 26: 1017–1025. 22382152 10.1097/QAD.0b013e328352d1ad PMC3580210

[7] Crothers K, Butt AA, Gibert CL, Rodriguez-Barradas MC, Crystal S, et al (2006) Increased COPD among HIV-positive compared to HIV-negative veterans. Chest 130: 1326–1333. 17099007 10.1378/chest.130.5.1326

[8] Drummond MB, Kirk GD, McCormack MC, Marshall MM, Ricketts EP, et al (2010) HIV and COPD: impact of risk behaviors and diseases on quality of life. Qual Life Res 19: 1295–1302. 20617387 10.1007/s11136-010-9701-x PMC4097077

[9] Polish LB, Cohn DL, Ryder JW, Myers AM, O'Brien RF (1989) Pulmonary non-Hodgkin's lymphoma in AIDS. Chest 96: 1321–1326. 2582839 10.1378/chest.96.6.1321

[10] Hoover DR, Saah AJ, Bacellar H, Phair J, Detels R, et al (1993) Clinical manifestations of AIDS in the era of pneumocystis prophylaxis. Multicenter AIDS Cohort Study. N Engl J Med 329: 1922–1926. 7902536 10.1056/NEJM199312233292604

[11] Gelman M, King MA, Neal DE, Pacht ER, Clanton TL, et al (1999) Focal air trapping in patients with HIV infection: CT evaluation and correlation with pulmonary function test results. AJR Am J Roentgenol 172: 1033–1038. 10587143 10.2214/ajr.172.4.10587143

[12] Jasmer RM, Edinburgh KJ, Thompson A, Gotway MB, Creasman JM, et al (2000) Clinical and radiographic predictors of the etiology of pulmonary nodules in HIV-infected patients. Chest 117: 1023–1030. 10767234 10.1378/chest.117.4.1023

[13] Hardak E, Brook O, Yigla M (2010) Radiological features of Pneumocystis jirovecii Pneumonia in immunocompromised patients with and without AIDS. Lung 188: 159–163. 20049469 10.1007/s00408-009-9214-y

[14] Sider L, Gabriel H, Curry DR, Pham MS (1993) Pattern recognition of the pulmonary manifestations of AIDS on CT scans. Radiographics 13: 771–784 discussion 785–776. 8356267 10.1148/radiographics.13.4.8356267

[15] Hartman TE, Primack SL, Muller NL, Staples CA (1994) Diagnosis of thoracic complications in AIDS: accuracy of CT. AJR Am J Roentgenol 162: 547–553. 8109494 10.2214/ajr.162.3.8109494

[16] Gold JA, Rom WN, Harkin TJ (2002) Significance of abnormal chest radiograph findings in patients with HIV-1 infection without respiratory symptoms. Chest 121: 1472–1477. 12006431 10.1378/chest.121.5.1472

[17] Sigel K, Wisnivesky J, Shahrir S, Brown ST, Justice A, et al (2014) Findings in asymptomatic HIV-infected patients undergoing chest computed tomography testing: implications for lung cancer screening. AIDS 28: 1007–1014. 24401647 10.1097/QAD.0000000000000189 PMC4018450

[18] George MP, Kannass M, Huang L, Sciurba FC, Morris A (2009) Respiratory symptoms and airway obstruction in HIV-infected subjects in the HAART era. PLoS One 4: e6328. 19621086 10.1371/journal.pone.0006328 PMC2709444

[19] Drummond MB, Kirk GD, Ricketts EP, McCormack MC, Hague JC, et al (2010) Cross sectional analysis of respiratory symptoms in an injection drug user cohort: the impact of obstructive lung disease and HIV. BMC Pulm Med 10: 27. 20459792 10.1186/1471-2466-10-27 PMC2876103

[20] Gingo M, Balasubramani G, Kingsley L, Kleenup EC, Seaberg E, et al.. (2012) HIV Infection and Prevalence of Pulmonary Disease: Multicenter AIDS Cohort Study. Conference on Retroviruses and Opportunistic Infections. Seattle, WA.

[21] Nakano Y, Muro S, Sakai H, Hirai T, Chin K, et al (2000) Computed tomographic measurements of airway dimensions and emphysema in smokers. Correlation with lung function. Am J Respir Crit Care Med 162: 1102–1108. 10988137 10.1164/ajrccm.162.3.9907120

[22] Yuan R, Hogg JC, Pare PD, Sin DD, Wong JC, et al (2009) Prediction of the rate of decline in FEV(1) in smokers using quantitative Computed Tomography. Thorax 64: 944–949. 19734130 10.1136/thx.2008.112433 PMC3035577

[23] Haruna A, Muro S, Nakano Y, Ohara T, Hoshino Y, et al (2010) CT scan findings of emphysema predict mortality in COPD. Chest 138: 635–640. 20382712 10.1378/chest.09-2836

[24] Gingo MR, Wenzel SE, Steele C, Kessinger CJ, Lucht L, et al (2012) Asthma diagnosis and airway bronchodilator response in HIV-infected patients. J Allergy Clin Immunol 129: 708–714 e708 . 22177327 10.1016/j.jaci.2011.11.015 PMC3294124

[25] Comstock GW, Tockman MS, Helsing KJ, Hennesy KM (1979) Standardized respiratory questionnaires: comparison of the old with the new. Am Rev Respir Dis 119: 45–53. 10.1164/arrd.1979.119.1.45 420437

[26] Hansell DM, Bankier AA, MacMahon H, McLoud TC, Muller NL, et al (2008) Fleischner Society: glossary of terms for thoracic imaging. Radiology 246: 697–722. 18195376 10.1148/radiol.2462070712

[27] Muller NL, Staples CA, Miller RR, Abboud RT (1988) "Density mask". An objective method to quantitate emphysema using computed tomography. Chest 94: 782–787. 3168574 10.1378/chest.94.4.782

[28] Miller MR, Hankinson J, Brusasco V, Burgos F, Casaburi R, et al (2005) Standardisation of spirometry. Eur Respir J 26: 319–338. 16055882 10.1183/09031936.05.00034805

[29] Hankinson JL, Odencrantz JR, Fedan KB (1999) Spirometric reference values from a sample of the general U.S. population. Am J Respir Crit Care Med 159: 179–187. 9872837 10.1164/ajrccm.159.1.9712108

[30] Neas LM, Schwartz J (1996) The determinants of pulmonary diffusing capacity in a national sample of U.S. adults. Am J Respir Crit Care Med 153: 656–664. 8564114 10.1164/ajrccm.153.2.8564114

[31] Jasmer RM, Gotway MB, Creasman JM, Webb WR, Edinburgh KJ, et al (2002) Clinical and radiographic predictors of the etiology of computed tomography-diagnosed intrathoracic lymphadenopathy in HIV-infected patients. J Acquir Immune Defic Syndr 31: 291–298. 12439204 10.1097/00126334-200211010-00004

[32] Kirk GD, Merlo C, O'Driscoll P, Mehta SH, Galai N, et al (2007) HIV infection is associated with an increased risk for lung cancer, independent of smoking. Clin Infect Dis 45: 103–110. 17554710 10.1086/518606 PMC4078722

[33] Engels EA, Brock MV, Chen J, Hooker CM, Gillison M, et al (2006) Elevated incidence of lung cancer among HIV-infected individuals. J Clin Oncol 24: 1383–1388. 16549832 10.1200/JCO.2005.03.4413

[34] Wilson DO, Weissfeld JL, Fuhrman CR, Fisher SN, Balogh P, et al (2008) The Pittsburgh Lung Screening Study (PLuSS): outcomes within 3 years of a first computed tomography scan. Am J Respir Crit Care Med 178: 956–961. 18635890 10.1164/rccm.200802-336OC PMC2720144

[35] National Lung Screening Trial Research T, Aberle DR, Adams AM, Berg CD, Black WC, et al (2011) Reduced lung-cancer mortality with low-dose computed tomographic screening. N Engl J Med 365: 395–409. 21714641 10.1056/NEJMoa1102873 PMC4356534

[36] National Lung Screening Trial Research T, Church TR, Black WC, Aberle DR, Berg CD, et al (2013) Results of initial low-dose computed tomographic screening for lung cancer. N Engl J Med 368: 1980–1991. 23697514 10.1056/NEJMoa1209120 PMC3762603

[37] Diaz PT, King MA, Pacht ER, Wewers MD, Gadek JE, et al (2000) Increased susceptibility to pulmonary emphysema among HIV-seropositive smokers. Ann Intern Med 132: 369–372. 10691587 10.7326/0003-4819-132-5-200003070-00006

[38] Wilson DO, Weissfeld JL, Balkan A, Schragin JG, Fuhrman CR, et al (2008) Association of radiographic emphysema and airflow obstruction with lung cancer. Am J Respir Crit Care Med 178: 738–744. 18565949 10.1164/rccm.200803-435OC PMC2556456

